# Analysis of Thyroid Imaging Reporting and Data System Criteria and Its Correlation With the Pathological Results

**DOI:** 10.7759/cureus.40117

**Published:** 2023-06-08

**Authors:** Vineel Sai Deepak Kallepalli, Thirugnanasambandam Nelson, Saravanan Sanniyasi

**Affiliations:** 1 General Surgery, Sri Ramachandra Institute of Higher Education and Research, Chennai, IND; 2 Surgery, Maulana Azad Medical College, New Delhi, IND; 3 Surgery, All India Institute of Medical Sciences, New Delhi, New Delhi, IND

**Keywords:** echogenic foci, acr tirads, malignancy, fnac, thyroid nodules

## Abstract

Introduction

Thyroid nodules are frequently encountered and may be discovered roughly in around 4-8% of individuals by clinical palpation.

Aim

The present study aims to analyze the Thyroid Imaging Reporting and Data Systems (TIRADS) classification and assess the validity of each criterion in predicting malignancy.

Methods

A prospective observational study was conducted at Sri Ramachandra Institute of Higher Education and Research from June 2020 to October 2021. Fifty patients who presented to the outpatient clinic with thyroid swelling got an ultrasound (USG) neck performed followed by either fine-needle aspiration cytology (FNAC) or thyroidectomy. They were included in the study and informed consent was obtained from all patients.

Results

Among 50 patients considered for the study, 36 were females. The mean age for malignant patients is 46 years with a standard deviation (SD) of 15, and for benign lesions is 47 years with an SD of 1. Most of the patients were TIRADS 4, which has a 56.2% risk of malignancy. The pathological results show a significant difference in ACR (American College of Radiology) TIRADS and echogenic foci between FNAC. The present study's solid composition showed a sensitivity of 25% and specificity of 75%, with an odds ratio of 0.90 in picking up malignant nodules. The shape of a nodule taller than wider, a malignant feature, showed a specificity of 92.3%. Punctate echogenic foci showed a sensitivity of 50% and specificity of 76.9% with a statistical significance of p-value 0.048.

Conclusion

TIRADS scoring avoids unessential invasive techniques for lower TIRADS scores. Certain criteria are more specific in recognizing malignant nodules. Proportional priority shall be given to certain criteria over others, and not all criteria should be considered.

## Introduction

Thyroid nodules are relatively common and may be discovered in roughly 4-8% of individuals by clinical palpation. However, the prevalence rate rises very high when imaging modalities such as high-resolution ultrasonography (USG) are used, ranging from 20% to 76% [1]. The vast majority of nodules do not cause any symptoms and are harmless; nonetheless, when they are found either by chance or clinically, they require a workup and further examinations. Because endocrine diseases are so prevalent in women, thyroid nodules are found in women 16 times more often than in men. The frequency of malignancy in thyroid nodules ranges from 5% to 10%. However, benign thyroid lesions are far more prevalent than their malignant counterparts [2,3].

In contrast to thyroid nodules, thyroid cancer is extremely uncommon, with an incidence rate of 8.7 per 100,000 persons yearly, although this rate appears to be growing over the years. With a sensitivity of over 90% and a specificity of 75%, fine-needle aspiration cytology (FNAC) has emerged as the most successful method for evaluating thyroid nodules in recent years [4]. Although FNAC is still an essential part of the diagnosis process for thyroid nodules, the procedure is a little unsettling, results in increased expenditures for medical treatment, and presents a marginal possibility of infection and bruising [5].

Investigating a thyroid nodule can also be accomplished using an ultrasound (USG), which is both non-invasive and affordable. Horvath et al. made the initial report on the findings of Thyroid Imaging Reporting and Data Systems (TIRADS) in 2009 [6]. According to their possibility of developing cancer, the various USG patterns of thyroid nodules have been categorized into distinct TIRADS classes using the data. It is generally agreed that USG-guided FNAC is the procedure that provides the best reliable assessment of thyroid cancer. However, a biopsy is not always an option because a significant proportion of thyroid nodules are benign, and the procedure is somewhat intrusive. As a result, USG is essential for distinguishing benign from malignant thyroid nodules and preventing needless intrusive operations in benign thyroid nodules [7-9].

Aim

To analyze the TIRADS classification and assess the validity of each criterion in predicting malignancy.

## Materials and methods

A prospective observational study was conducted at Sri Ramachandra Institute of Higher Education and Research from June 2020 to October 2021. Fifty patients with thyroid swelling got USG neck performed followed by either FNAC or thyroidectomy. They were included in the study and informed consent was obtained from all patients for the same.

Inclusion criteria

Patients with thyroid swellings undergoing a preoperative USG with TIRADS grading, age group between 18 and 80 years, and patients with thyroid swellings undergoing FNAC or thyroidectomies were included.

Exclusion criteria

Patients with thyroid swelling who did not undergo any pathological evaluation and those with USG evaluation but no individual TIRADS scoring were excluded.

Methodology

All the necessary clinical data, USG details, and TIRADS category were recorded in the proforma prepared for this study. In addition, after the completion of FNAC or thyroid surgery, the pathological result of the FNAC or final histopathological biopsy report was collected and recorded in the proforma. Evaluation of thyroid lesions using B-Mode sonography and Doppler was carried out. An evaluation was performed on both lobes of the thyroid gland in addition to examining the isthmus. After placing the patient in a supine posture and putting their neck in a hyperextended position, the whole gland was checked. Because of its relatively superficial position, the thyroid could be imaged sonographically to reveal even minute anatomical alterations.

Sagittal, transverse, and oblique portions of the neck were scanned to get excellent visualization of both the thyroid lobes and isthmus as well. The scan also checked for any concomitant cervical lymphadenopathy. The thyroid nodules, if present, were staged according to TIRADS classification. First, the ACR (American College of Radiology) TIRADS considers the following five feature criteria when evaluating a sample: composition, echogenicity, shape, margin, and echogenic foci. Then score was assigned according to the criteria met to get the total TIRADS score and then the TIRADS grade accordingly. Next, the patient underwent FNAC of the thyroid nodule to establish the pathological result. Next, the cytological findings were evaluated using the Bethesda System for Reporting Thyroid Cytopathology and categorized accordingly. Finally, the choice of surgery performed was made by the clinician based on the USG and the FNAC reports and followed up for the final histopathological report.

The Institutional Research Ethics Committee, Sri Ramachandra Institute of Higher Education and Research issued the approval for conducting this study with the IRB number CSP-MED/20/JUN/COVID/04.

## Results

The results have been tabulated to portray the outcome of the study. Table 1 depicts the demographics of the present study population along with their histopathological outcome. 

**Table 1:** Demographic data of the study TIRADS, Thyroid Imaging Reporting and Data Systems.

Variable		Benign	Malignant
Gender	Male	10	4
	Female	16	20
Age	18-30	3	4
	31-60	19	13
	>60	4	7
TIRADS	2	5	2
	3	9	3
	4	10	11
	5	2	6

Among the 50 patients, the majority (36) were females and 14 were males. The mean age of malignant patients is 46 years with a standard deviation (SD) of 15, and for benign lesions is 47 years with an SD of 14. Most of the patients were TIRADS 4 category which has a 56.2% risk of malignancy. Table 2 compares the TIRADS grade and each criterion separately with the final histopathological outcome.

**Table 2:** Each criterion of TIRADS and pathological results of the study ACR TIRADS, American College of Radiology Thyroid Imaging Reporting and Data Systems; FNAC, fine-needle aspiration cytology; ETE, extra thyroidal extension.

Variable		FNAC		p-Value
		Benign	Malignant	
ACR TIRADS	2 and 3	5 (20.8%)	14 (53.8%)	0.016
	4 and 5	19 (79.2%)	12 (46.2%)	
Composition	Solid	6 (25%)	7 (26%)	0.877
	Mixed/Cystic/Sponge	18 (75%)	19 (73%)	
Echogenicity	Hypo/ Very Hypo	12 (50%)	9 (34.6%)	0.271
	Hyper/Iso/Anechoic	12 (50%)	17 (65.4%)	
Shape	Taller than wide	3 (12.5%)	2 (7.7%)	0.661
	Wider than tall	21 (87.5%)	24 (92.3%)	
Margin	Irregular/ETE	6 (25%)	2 (7.7%)	0.132
	Smooth	18 (75%)	24 (92.3%)	
Echogenic foci	Micro/ Punctate/Speck	12 (50%)	6 (23.1%)	0.048
	None/Macro/Peripheral rim/Comet tail	12 (50%)	20 (76.9%)	

The results showed a significant difference between ACR TIRADS grade and FNAC (p=0.016). But FNAC had no significant difference in composition, echogenicity, shape, and margin. Keeping the echogenic foci TIRADS criteria into consideration, the authors intend to state the statistically significant difference in FNAC outcomes with p values =0.048 (benign vs malignant) between TIRADS 2 and 3 and TIRADS 4 and 5. The other criterion could not achieve a statistically significant difference. Table 3 displays the statistical results of the study variables with their outcomes.

**Table 3:** Results of all TIRADS criteria in the study USG, ultrasound; PPV, positive predictive value; NPV, negative predictive value; ETE, extra thyroidal extension; TIRADS, Thyroid Imaging Reporting and Data Systems.

USG feature	Sensitivity (%)	Specificity (%)	PPV (%)	NPV (%)	Accuracy (%)	Odds ratio	p-Value
Composition (solid)	25	73.1	46.2	51.4	50	0.90	0.877
Echogenicity (hypo and very hypoechoic)	50	65.4	57.1	58.6	58	1.89	0.271
Shape (taller than wide)	12.5	92.3	60	53.3	54	1.57	0.661
Margins (irregular/ ETE)	25	92.3	75	57.1	60	3.63	0.132
Echogenic foci (punctate microcalcifications)	50	76.9	66.7	62.5	64	4.33	0.048

The present study's solid composition showed a sensitivity of 25% and specificity of 75%, with an odds ratio of 0.90 in picking up malignant nodules. The shape of a nodule taller than wider, a malignant feature, showed a specificity of 92.3%. Punctate echogenic foci showed a sensitivity of 50% and specificity of 76.9% with a statistical significance of p-value 0.048.

## Discussion

Thyroid swellings are a common entity and have been thought to be seen in around 12% of the Indian population by Usha Menon et al. [10]. There is a lower risk of cancer developing within these lesions overall. USG is more sensitive than palpation and can detect nodules that are too small to be palpated. According to Ko et al. [11], ACR TIRADS had the largest area under the receiver operating characteristic (ROC) curve when it came to identifying nodules with a cytologically high risk.

In a study by Xu et al. [12] ACR TIRADS showed the best sensitivity (96.9%) and lowest specificity (52.9%), and the area under the curve (AUC) of the ROC curve was slightly lower than the Korean Society of Thyroid Radiology (KSThR). Furthermore, the rate of unnecessary FNAC was lowest with ACR TIRADS (17.3%), followed by European Thyroid Association TIRADS (EU-TIRADS; 25.2%) and KSThR TIRADS with 42.6%, which were found to be statistically significant in their study. On validation of the ACR TIRADS in our study, we discovered that the risk of malignancy increased progressively as the TIRADS score increased from 2 to 5, as originally proposed by Horvath et al. [6]. Compared with other studies, Srinivas et al. [13] discovered a 100% malignancy risk in TIRADS 5. In comparison, Kwak et al. [14] found a malignancy risk of 87.5% in TIRADS 5, and our study showed a malignancy risk of 75% in TIRADS 5.

Another validation study of TIRADS by Horvath et al. [15] showed a similar trend in picking the malignant nodule percentage. In their validation study, a cutoff value of TRADS 4 for malignancy yielded a sensitivity of 99.6% and a specificity of 74.35%. This corroborated with Zhang et al. [16], where the cutoff was TIRADS 5 with a sensitivity of 81.4% and a specificity of 84.8%. Xu et al. [17] also reported a similar cutoff for TIRADS 4 with a sensitivity of 80.4% and a specificity of 79%.

Therefore, we also applied the same cutoff for malignant nodules in our study and found that TIRADS 4 and 5 were picking up malignant nodules with a sensitivity of 79.2%, a specificity of 53%, an accuracy of 66%, and a p-value of 0.016 which is statistically significant. In our study, solid composition showed a sensitivity of 25% and a specificity of 75%, with an odds ratio of 0.90 in picking up malignant nodules. This is much less compared to those in the studies by Srinivas et al. [13] and Kwak et al. [14], which portrayed 10 times the odds ratio derived from this study. This could be attributed to the discordant sample pool and a small sample size compared to the other studies.

The shape of a nodule taller than wider being a malignant feature showed a specificity of 92.3% in our study, similar to the findings from Srinivas et al. [13] and Patil et al. [18] and their studies. Hence, we can say that this feature will be associated with a malignant pathology, but in our study, this finding failed to achieve a statistically significant value. On the other hand, the margins of the nodule being irregular or extrathyroidal extension makes us interpret them as malignant, as proven by Srinivas et al. [13] who found it to be highly specific - 99.71%. Similarly, our study has a specificity of 92.3% with a p-value of 0.132.

In this study, punctate echogenic foci showed a sensitivity of 50% and a specificity of 76.9%, with a statistically significant p-value of 0.048. This resembled the findings from Srinivas et al. [13] and Patil et al. [18], that is, a specificity of 98.5% and 98.8%, respectively. Echogenicity was found to have the lowest degree of agreement (=0.34) of all the USG characteristics among the experienced radiologists who had worked in the field for more than five years, according to Choi et al. [19]'s findings. Kim et al. [20] also showed that echogenicity is one of the inexperienced radiologists' and residents’ most subjective USG features. This variation in training can be the reason for differences among the sonologists in our institute, resulting in the incongruencies of the final results in our study with very hypoechoic lesions also ending up having benign pathology. Choi et al. [19] reported that the κ value of experienced radiologists was 0.53 for well-defined margins, 0.35 for micro-lobulated margins, and 0.23 for irregular or spiculated margins.

This is challenging for both residents and the experienced to distinguish between micro-lobulated and irregular borders of thyroid nodules. In our study, we found that most of the nodules had smooth margins but, on the other hand, the number of nodules with ill-defined or irregular margins is less to show these variations. And all the more, nodules with these findings became malignant because of the coexistence of other suspicious findings.

The subjective nature of USG per se would be counted as a limitation of this study accounting for the discrepancies in the results it has on being compared with other studies. Apart from this, the sample population included is too small to extrapolate the findings to larger population-based studies compared within the discussion.

## Conclusions

This study substantiates the usefulness of TIRADS scoring and avoids unessential invasive techniques for lower TIRADS scores. These results were supplemented with the ACR TIRADS consensus reports. In addition, an analysis of various parameters in TIRADS highlighted that certain criteria were more specific in recognizing malignant nodules. Hence, proportional priority shall be given to certain criteria over others. A study with a larger population would substantiate these findings.
